# A Hybrid Federated Learning Framework for Enhancing Privacy and Robustness in Non-Intrusive Load Monitoring

**DOI:** 10.3390/s26020443

**Published:** 2026-01-09

**Authors:** Jing Rong, Qiuzhan Zhou, Huinan Wu

**Affiliations:** College of Communication Engineering, Jilin University, Changchun 130012, China

**Keywords:** smart-grid advanced metering infrastructure, non-intrusive load monitoring (NILM), privacy preservation, federated learning, model robustness

## Abstract

Non-intrusive load monitoring (NILM), as a key technology in smart-grid advanced metering infrastructure, aims to disaggregate mains power from smart meters into individual load-level power consumption. Traditional NILM methods require centralizing sensitive measurement data from users, which poses significant privacy risks. Federated learning (FL) enables collaborative training without centralized measurement data, effectively preserving privacy. However, FL-based NILM systems face serious threats from attacks such as model inversion and parameter poisoning, and rely heavily on the availability of a central server, whose failure may compromise measurement robustness. This paper proposes a hybrid FL framework that dynamically switches between centralized FL (CFL) and decentralized FL (DFL) modes, enhancing measurement privacy and system robustness simultaneously. In CFL mode, layer-sensitive pruning and robust parameter aggregation methods are developed to defend against model inversion and parameter poisoning attacks; even with 30% malicious clients, the proposed defense limits the increases in key error metrics to under 15.4%. In DFL mode, a graph attention network (GAT)-based dynamic topology adapts to mitigate topology poisoning attacks, achieving an approximately 17.2% reduction in MAE after an attack and rapidly restoring model performance. Extensive evaluations using public datasets demonstrate that the proposed framework significantly enhances the robustness of smart-grid measurements and effectively safeguards measurement privacy.

## 1. Introduction

With the continuous growth of global energy demand and the advancement of carbon-neutral goals, efficient energy management in smart grids becomes a crucial research topic [1]. Advanced metering infrastructure, as a vital component of smart grids, enables measurement and analysis of user-side electricity consumption data, thereby promoting grid intelligence and interactivity. Non-intrusive load monitoring (NILM) is a core technology within advanced metering infrastructure. It analyzes aggregate mains power measured by smart meters to infer individual load power and identify electricity usage patterns [2]. Consequently, NILM facilitates effective user behavior analysis, energy management, and demand-response services [3].

From the perspective of technological evolution, early NILM mainly relied on traditional approaches such as event detection and hidden Markov models. Subsequently, deep learning methods, including convolutional neural networks (CNNs) [4,5] and recurrent neural networks (RNNs) [6], substantially improved the disaggregation accuracy for complex loads by learning temporal representations in an end-to-end manner. In recent years, global-dependency models such as Transformers [7] have further strengthened the capability of load disaggregation for long sequences and multi-scale patterns, and they are progressively combined with transfer learning [8], reinforcement learning [9], and closed-loop calibration mechanisms [10], thereby effectively improving model generalization and robust disaggregation performance for multi-state loads. At the application level, studies on distribution network operation and home energy management based on smart meter data also indicate that NILM provides key inputs for flexible resource scheduling, demand response, and network operational support, thereby promoting measurement-driven monitoring and operational decision-making [11,12,13].

However, traditional NILM methods rely on centralized model training, which requires collecting large volumes of smart meter measurement data. This centralized approach significantly increases the risk of exposing sensitive user information (e.g., usage patterns) and violates strict privacy regulations such as the European Union’s General Data Protection Regulation (GDPR) [14]. Consequently, privacy concerns hinder the widespread adoption of NILM in practical smart grid scenarios. This may limit the availability of fine-grained consumption insights that support measurement-driven monitoring and operational decision-making in smart grids [15,16].

To mitigate these privacy issues, federated learning (FL) emerges as a promising learning paradigm. FL enables multiple clients to train models locally while sharing only model parameters or gradients instead of raw measurement data with a server or peer clients, fundamentally avoiding the transmission and central storage of sensitive data. This approach significantly reduces privacy leakage risks for NILM applications [17]. Depending on the communication architecture, FL has two main modes: centralized FL (CFL) and decentralized FL (DFL). CFL uses a central server to coordinate global model aggregation and parameter distribution, with communication only between the server and clients [18]. This mode is simple, efficient, and widely adopted in practice. DFL eliminates the server; clients exchange model parameters directly via peer-to-peer communication to achieve collaborative learning [19].

Although some studies apply FL to NILM tasks [20], they mostly assume ideal conditions and overlook diverse attack threats present in open smart grids (e.g., model inversion [21,22], parameter poisoning [23], topology poisoning [24]). Existing works propose defense techniques targeting specific attacks, such as incorporating differential privacy [25] or employing robust aggregation algorithms [26], but these approaches still suffer from several notable limitations. (i) Lack of integration between privacy and robustness: Methods like differential privacy [25] and homomorphic encryption [27] enhance privacy but often ignore active attack scenarios, lacking synergy with robust defenses. (ii) Single-purpose defenses: Most solutions focus on one attack type (e.g., Byzantine faults [28] or backdoors [29]) and fail under coordinated or multi-stage attacks. Stealthy distributed attacks can evade detection, leaving defenses fragmented and inefficient. (iii) Static DFL topologies: DFL typically uses fixed communication topologies [30]. A malicious client at a key position can spread poisoned updates unchecked, and the lack of dynamic reconfiguration makes isolation and recovery difficult.

To address these challenges, we propose a hybrid FL framework that integrates measurement privacy-preservation with robust defense mechanisms. The framework can dynamically switch between CFL and DFL modes based on the central server’s status, and it incorporates tailored defenses for specific attack scenarios. Note that this work builds upon our previously proposed NILM model, TransDisNILM [8]. The main contributions of this framework are as follows:

(i) CFL mode defenses: We introduce a layer-sensitive pruning strategy for federated updates in smart grid load monitoring, which reduces model parameter invertibility to preserve the privacy of power system measurement data against inversion attacks. In addition, a robust aggregation method with filtering and fine-tuning is applied to defend against parameter poisoning attacks, ensuring the accuracy of load disaggregation measurements.

(ii) DFL mode defenses: If the central server fails or is compromised, the framework automatically switches to DFL mode, employing a graph attention network (GAT)-based dynamic communication topology adaptation along with proactive defenses. Periodic topology reconstruction prevents the continued spread of poisoned parameters, greatly enhancing measurement robustness under DFL.

(iii) Validated effectiveness: Extensive simulations on NILM scenarios demonstrate that our hybrid FL framework markedly improves NILM accuracy and system resilience under difference attacks. The results confirm the framework’s effectiveness in practical smart grid deployments.

The rest of this paper is organized as follows: Section 2 reviews related work on NILM model and FL security. Section 3 defines the NILM problem and attack models in an FL setting. Section 4 details the proposed hybrid FL framework and its defense methods. Section 5 presents the simulation evaluation results. Section 6 concludes the paper and discusses future work.

## 2. Related Work

### 2.1. Advances in NILM Algorithms and Applications

NILM aims to disaggregate loads from aggregate power measurements, providing crucial support for demand-side energy management in smart grids. In recent years, deep learning models such as CNN [4,5], RNN [6], and attention-based architectures [7] have significantly improved load disaggregation performance by efficiently learning high-dimensional temporal features. Furthermore, Transformer architectures [8], due to their strong capability for modeling long-range dependencies, are adopted to improve disaggregation accuracy and cross-scenario generalization. Meanwhile, closed-loop learning [10] and reinforcement-learning-based strategies [9] continuously enhance disaggregation accuracy through error feedback or policy optimization mechanisms, which are especially suitable for multi-state loads and complex operating patterns. From an application perspective, studies on distribution-network and household-side applications based on smart-meter data show that the fine-grained load observability provided by NILM supports tasks such as demand response, flexible resource scheduling, and distribution-network operation assessment, thereby extending NILM from energy disaggregation toward measurement-driven monitoring and operational decision support [11,12,13]. However, these deep learning approaches typically rely on centralized training with large amounts of user measurement data, which leads to serious privacy risks and difficulties in complying with regulations like GDPR [14].

### 2.2. Federated Learning for Privacy-Preserving NILM

In AMI scenarios, high-resolution electricity consumption measurements are highly privacy-sensitive. Centralized data collection and long-term storage often face compliance constraints and barriers to data sharing, and they are particularly difficult to reconcile with privacy regulations such as the GDPR, which impose requirements on data collection, purpose limitation, and minimization principles. Therefore, even when the model design and application demand are clear, a centralized training pipeline on the data side may still be constrained in practical deployments.

To address the privacy concerns of centralized training, FL is introduced into NILM. In an FL system, clients train models locally and share only model parameters or gradients with a central server, thus avoiding the transmission of raw user measurement data. For example, WANG et al. [20] first apply a federated averaging algorithm to NILM, demonstrating that FL can preserve measurement privacy in load disaggregation. DAI et al. [25] further incorporate differential privacy by adding Gaussian noise to model parameters during aggregation, significantly reducing potential data leakage.

Overall, existing FL-based NILM studies validate the feasibility of keeping data local and achieve progress in privacy enhancement and personalized training. However, current FL system settings often assume ideal conditions, such as stable communication, protocol-abiding participants, and the absence of active adversarial behaviors. As a result, the discussion of security risks and robustness guarantees in open networks and unreliable environments remains relatively limited.

### 2.3. Robust Federated Learning Under Unreliable Environments

Recent studies indicate FL’s vulnerability to multiple attacks, prompting the development of defense methods against model inversion attacks, parameter poisoning attacks, and topology poisoning attacks:

(i) Model inversion attacks aim to reconstruct sensitive client data from shared parameters or gradients, posing severe privacy threats. Zhu et al. [22] propose gradient compression and noise confusion techniques to defend against gradient leakage. Li et al. [21] introduce a generative adversarial network (GAN)-based inversion attack, highlighting the insufficiency of traditional noise injection against high-quality inversion attacks. Recent studies reveal that parameter compression significantly reduces sensitive associations within model parameters. This approach effectively mitigates model inversion attacks [22,31]. Nevertheless, these methods inadequately balance model performance and measurement privacy-preservation, leading to significant performance degradation at high compression levels [31].

(ii) Parameter poisoning attacks, including destructive Byzantine attacks and backdoor attacks, manipulate FL models through malicious parameter uploads. Robust aggregation algorithms such as multi-Krum [28], FLAME [26], and trimmed mean [32] employ statistical methods to identify and exclude anomalous client parameters, effectively mitigating malicious impacts. However, these methods typically assume a low proportion of malicious clients, significantly degrading performance under large-scale or coordinated Byzantine attacks. Recent studies enhance Byzantine robustness from the joint perspective of authentication and aggregation by using adaptive authentication mechanisms to improve robustness bounds [33]. In addition, other studies propose random-matching verification and reputation-table-based aggregation frameworks to maintain update credibility in unreliable environments [34]. Against stealthier backdoor attacks, existing studies mainly rely on abnormal sample detection methods [35], yet these approaches inadequately address subtle parameter-level backdoor injections.

(iii) Topology poisoning attacks: In DFL, attackers may forge neighbor connections to propagate poisoned model parameters across the peer-to-peer network, severely degrading model performance. Most existing DFL studies use fixed communication topologies (e.g., fully connected or ring-connected networks [30]), which are susceptible to long-term infiltration by adversaries.

To tackle topology attacks, some studies explore dynamic or hybrid FL solutions. Reference [24] proposes a blockchain-based identity verification mechanism to detect malicious clients. However, this approach incurs high communication and computation overhead, limiting its practical deployment in smart grid measurement systems. Reference [36] suggests pre-defined rules to trigger a switch to DFL mode when an attack is detected, but it lacks detailed considerations of measurement privacy-preservation in the new mode.

## 3. Problem Formulation

### 3.1. NILM Multi-Load Power Disaggregation

Let the aggregated mains power at time *t* be denoted by p(t). Suppose the system has *L* loads, and let $p_{l}\left(t\right)$ represent the power consumption of the *l*-th load (l=1,…,L). We denote by n(t) the noise component at time *t*. Based on these definitions, the aggregate mains power can be expressed as [15]:

(1) $$p\left(t\right)=\sum_{l=1}^{L}p_{l}\left(t\right)+n\left(t\right).$$

Given a sequence of aggregate power with window length *w* (an odd integer), $p\left(t\right)={\left[p\left(t-\frac{\left(w-1\right)}{2}\right),\ldots ,p\left(t+\frac{\left(w-1\right)}{2}\right)\right]}^{⊤}\in R^{w}$. The NILM model f(·;Θ) is trained to minimize the disaggregation error. The objective is to find model parameters Θ that minimize the mean squared error (MSE) between the disaggregated and actual power:

(2) $$\Theta^{*}=\arg\min_{\Theta }\frac{1}{\left|D\right|}\sum_{t\in D}\ell \left(f\left(p(t);\Theta \right),p_{l}\left(t\right)\right),$$

where ℓ(·,·) denotes the MSE loss, and |D| is the number of training samples.

However, in practice it is often infeasible to centralize all users’ measurement data for training due to privacy restrictions. This necessity motivates the use of FL for NILM (as described next) to enable privacy-preserving collaborative learning on distributed measurement data.

### 3.2. NILM Federated Learning and Robustness Defense

In an FL environment, data is distributed across *C* clients. Each client *c* holds a local dataset $D_{c}$. Model updates are collaboratively aggregated. This distributed setting introduces notable security threats. These threats include model inversion, parameter poisoning, and topology poisoning attacks, which can severely degrade model accuracy and robustness. In this paper, we define robustness as the capability of an FL system to maintain model performance and stable training in the presence of such attacks, effectively mitigating the impact of malicious clients.

Thus, we propose a hybrid FL framework that primarily operates in the CFL mode by default. If the central server fails or becomes unreliable, the framework can automatically transition to the DFL mode, thereby ensuring continuous collaborative learning on the smart grid measurement data. In each mode, we implement dedicated robustness enhancement methods to secure the training process. Next, we detail the training objectives and defense strategies for each mode.

#### 3.2.1. CFL Mode

All clients perform local training on their data and send model updates to the central server for global aggregation. The learning objective in CFL mode is:

(3) $$\Theta^{*}=\arg\min_{\Theta }\sum_{c=1}^{C}\omega_{c}F_{c}\left(\Theta \right),$$

where $\omega_{c}=\frac{|D_{c}|}{\sum_{j=1}^{C}\left|D_{j}\right|}$ is the weight of client *c*, and $F_{c}\left(\Theta \right)=\frac{1}{\left|D_{c}\right|}\sum_{t\in D_{c}}\ell \left(f\left(p(t);\Theta \right),p_{l}\left(t\right)\right)$ is the loss of client *c*. In CFL mode, we defend against the following attacks.

(i) Model inversion attack: Let the uploaded parameters be denoted as $\Theta =[\theta_{1},\ldots ,\theta_{m},\ldots ,\theta_{M}]$ where *M* is the number of layers in the NILM model. Given the intercepted parameters (or a subset of layers), an adversary attempts to reconstruct the client’s private training data. This attack is formulated as $\hat{D}=A\left(\theta_{m}\right)$, where A(·) denotes an inversion procedure operating on the observed uploaded parameters.

(ii) Parameter poisoning attack: A malicious client uploads a perturbed model parameter vector instead of $\Theta_{c}^{e}$. We write ${\tilde{\Theta }}_{c}^{e}=\Theta_{c}^{e}+\Delta_{c}$, where $\Delta_{c}$ is the adversarial perturbation. In this paper, we consider two representative cases. For the Byzantine attack [37], we let $\Delta_{c}=\Lambda_{c}$, where $\Lambda_{c}$ denotes a perturbation matrix with the same dimension as the model parameters, and each element ${\left(\Lambda_{c}\right)}_{ij}\neq 0$ with $|{\left(\Lambda_{c}\right)}_{ij}|$ takes a large value. For the backdoor attack [29], we set $\Delta_{c}=B_{c}⊙\Lambda_{c}$, where $B_{c}$ is a binary selection matrix with the same dimension as the model parameters (elements are only 0 or 1), an element of 0 indicates no perturbation, whereas 1 indicates perturbation. The operator ⊙ denotes element-wise multiplication.

#### 3.2.2. DFL Mode

When the central server is unavailable, clients switch to the DFL mode. Each client *c* exchanges model parameters with its neighbor clients and updates its model based on both its own and its neighbors’ information. The learning objective for each client *c* can be formulated as:

(4) $$\Theta_{c}^{*}=\arg\min_{\Theta_{c}}\frac{1}{|N_{c}|+1}\sum_{u\in N_{c}\cup \left\{c\right\}}F\left(\Theta_{u}\right),$$

where $|N_{c}|$ represents the number of neighbors of client *c*, and $F\left(\Theta_{u}\right)=\frac{1}{\left|D_{u}\right|}\sum_{t\in D_{u}}\ell \left(f\left(p\left(t\right);\Theta_{u}\right),p_{l}\left(t\right)\right)$ is the local loss of client *u*. In DFL mode, besides the above two attacks, we further consider topology poisoning attack. Specifically, an adversary manipulates the neighbor set of client *c* from $N_{c}$ to an attacked set ${\tilde{N}}_{c}$ (e.g., by injecting malicious neighbors), thereby increasing the probability that client *c* receives poisoned parameters ${\tilde{\Theta }}_{u}^{e}$ from $u\in {\tilde{N}}_{c}$. The attack objective is to facilitate the propagation of poisoned parameters through peer-to-peer exchanges by altering the effective communication neighborhood.

## 4. Hybrid FL Framework and Robust Defense

**Figure 1:**
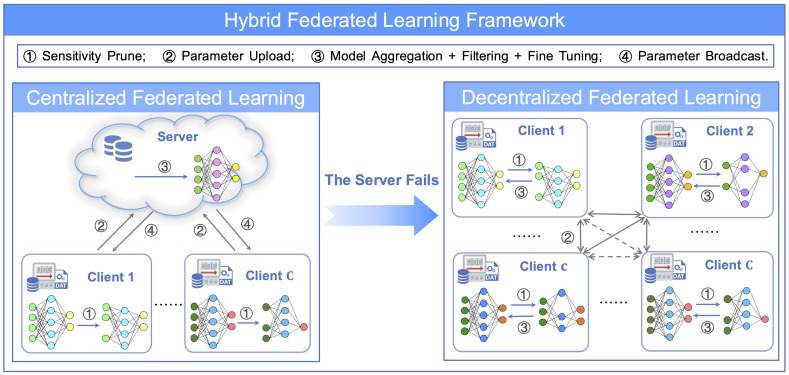
Hybrid federated learning framework.

To improve resilience against adversarial threats, we propose a hybrid FL framework that dynamically switches between CFL and DFL modes based on central server availability. The system operates in CFL mode by default, where a central server aggregates model updates. A heartbeat mechanism continuously monitors the central server status. If consecutive heartbeat responses are missed, indicating potential server failure or malicious attacks, the system automatically transitions to DFL mode, activating peer-to-peer communication among clients. Once the central server recovers, clients synchronize their models back to the central server and resume training in CFL mode. This design decision closely reflects realistic smart grid operations requiring robustness against central server failures or unstable network conditions. Figure 1 illustrates the hybrid FL architecture.

Specifically, the proposed model’s mode switching mechanism and triggering conditions are as follows:

(i) CFL→DFL Switch: A periodic heartbeat mechanism (every 10 training rounds) is adopted. Clients send heartbeat packets to the server. If no response (parameter broadcast or heartbeat ack) is received for 3 consecutive times, server failure is diagnosed, triggering switching: Clients establish initial peer-to-peer connections via preconfigured seed nodes, and the GAT-based dynamic topology initialization within one round. Local models retain their latest states as initial parameters for DFL. To avoid misclassifying transient network congestion as a server failure, the CFL→ DFL switching mechanism adopts a conservative multi-interval decision rule. A transition from CFL to DFL is triggered only when a client does not receive any global parameter broadcast or ACK from the server over several consecutive heartbeat periods and this phenomenon is observed by the majority of clients, so that sporadic packet loss or short-term link jitter does not cause spurious mode switching.

(ii) DFL→CFL Switch: Upon recovery, the server sends a timestamped recovery notification and global model snapshot to all clients: Clients stop peer-to-peer communication and update local models via incremental synchronization (only transmitting parameters differing from the server snapshot). The server restarts CFL training after confirming ≥90% of clients are synchronized. Symmetrically, in the DFL→CFL recovery phase, the server re-enables CFL training only after it has stably broadcast recovery notifications and the latest global model to the clients over several consecutive periods following recovery, and has verified that the majority of clients have synchronized to this snapshot, thereby preventing frequent back-and-forth switching when the network condition fluctuates around the connectivity threshold.

This framework ensures continuous federated collaboration for smart grid load monitoring. It significantly improves the robustness, fault tolerance, and reliability of power system measurements and operations.

### 4.1. Robustness Enhancement Methods in the CFL Mode

In CFL mode, the model faces risks of model inversion and parameter poisoning attacks. To enhance robustness, we introduce a combination of defenses: layer-sensitivity pruning, robust aggregation, filtering, and fine-tuning.

#### 4.1.1. Layer-Sensitivity Pruning for Model Inversion Attack Defense

In CFL mode, model inversion attacks allow adversaries to reconstruct local data by analyzing uploaded model parameters over iterations. The success of inversion attacks primarily depends on the richness of data representations embedded in the model parameter, particularly the invertibility of the complete parameter space. Therefore, reducing the invertibility of model parameters is one of the key strategies to mitigate model inversion attacks and preserve the underlying measurement data privacy.

Existing parameter pruning methods have notable limitations. Most methods only reduce transmitted parameters without evaluating their importance for accuracy or privacy risks. As a result, naive pruning can accidentally remove critical model parameters (degrading accuracy) while failing to significantly improve measurement privacy. As a solution, we propose a layer-sensitivity pruning strategy. This approach quantifies the importance of each layer’s parameters to the model’s performance, and then selectively uploads only the most important layers to the server. By transmitting only high-sensitivity layers, the scheme strikes a balance between preserving model measurement privacy and model accuracy.

First, we define a layer sensitivity metric $\delta_{m,c}^{e}$ to measure the importance of layer *m* for client *c* in round *e*. Specifically, it $\delta_{m,c}^{e}$ is defined as the absolute change in the layer’s mean parameter value between two training rounds: a large change indicates that layer *m* had a significant impact in that round.

(5) $$\delta_{m,c}^{e}=\left|\mathrm{mean}\left(\theta_{m,c}^{e}\right)-\mathrm{mean}\left(\theta_{m,c}^{e-1}\right)\right|.$$

Next, we rank all layers by $\delta_{m,c}^{e}$ and select the top aM layers (where 0<a<1 is a pruning ratio) as the high-sensitivity layer set $M_{c}^{e}$. Each client then uploads only the parameters ${\hat{\Theta }}_{c}^{e}=\left\{\theta_{m,c}^{e}|m\in M_{c}^{e}\right\}$ of layers in $M_{c}^{e}$ to the server. This way, only the most informative parameters are shared, which greatly reduces exposed sensitive measurement information.

It should be emphasized that the proposed layer-sensitive pruning is not intended to provide a strict privacy budget such as differential privacy. Rather, it limits the number of transmitted parameters observable to an adversary. By reducing the observable parameter dimensionality, it weakens the parameter-to-data invertibility and makes $\hat{D}=A\left(\theta_{m,c}^{e}\right)$ more likely underdetermined. Specifically, from a mathematical perspective, the above procedure is equivalent to applying a projection operator $\Pi_{M_{c}^{e}}$ to the full parameter set $\Theta_{c}^{e}$, i.e., ${\hat{\Theta }}_{c}^{e}=\Pi_{M_{c}^{e}}\left(\Theta_{c}^{e}\right)$. When $\left|M_{c}^{e}\right|<M$, this projection mapping is necessarily non-injective: there exist infinitely many distinct $\Theta_{c}^{e}$ that take different values on the non-uploaded layers but are identical on the uploaded layers, and therefore yield the same ${\hat{\Theta }}_{c}^{e}$. Formally, for any perturbation ΔΘ satisfying $\Delta \theta_{m}=0$, $\forall m\in M_{c}^{e}$ and $\Delta \theta_{m}\neq 0$ for some $m\notin M_{c}^{e}$, we have

(6) $$\Pi_{M_{c}^{e}}\left(\Theta_{c}^{e}+\Delta \Theta \right)=\Pi_{M_{c}^{e}}\left(\Theta_{c}^{e}\right)={\hat{\Theta }}_{c}^{e}$$

which implies that an adversary cannot uniquely determine the full $\Theta_{c}^{e}$ solely from ${\hat{\Theta }}_{c}^{e}$, and the inversion problem shifts from being determined to underdetermined. Moreover, this underdeterminedness can be directly quantified by the dimensionality of the hidden parameters. Let $d_{m}$ denote the parameter dimension of layer *m*. Then the hidden degrees of freedom associated with the non-uploaded layers are $d_{\mathrm{hide}\;}=\sum_{m\notin M_{c}^{e}}d_{m}$, meaning that, without changing ${\hat{\Theta }}_{c}^{e}$, there exist at least $d_{\mathrm{hide}}$ degrees of freedom that can vary.

Therefore, the mitigation effect of layer-sensitive pruning against inversion attacks is not merely empirical: when $\left|M_{c}^{e}\right|$ is smaller (i.e., a larger portion of layer parameters is not uploaded), the adversary observes less information and faces a larger feasible solution space, and the reconstruction difficulty increases mechanistically with $d_{\mathrm{hide}}$, thereby mathematically reducing the invertibility from parameters to data. After layer-sensitive pruning, each client transmits parameter updates only on the support set of its mask, so the upload dimensionality is reduced from the full model size *P* to (1−a)P. All subsequent robust aggregation and GAT-based topology construction on the server side are performed within this compressed parameter subspace.

During aggregation, the server performs joint aggregation only on the received high-sensitivity layer parameters. For unuploaded low-sensitivity layers, the server retains their values from the current global model to maintain model integrity. After receiving the global model, clients update their local high-sensitivity layers with aggregated parameters, ensuring consistency between local and global models.

#### 4.1.2. Robust Aggregation Against Byzantine Attacks

In an FL system, malicious clients can launch parameter poisoning attacks, for example, by injecting false updates to perform a Byzantine attack or embedding trigger patterns to execute a backdoor attack. To counter the aforementioned threats effectively, we propose a comprehensive defense strategy comprising: robust aggregation of model updates, data-level filtering, and server-side fine-tuning.

Specifically, we integrate multi-Krum [28] and trimmed mean [32] algorithms to robustly aggregate server parameters, defending against Byzantine attacks. By combining these methods, our approach performs screening of model updates to detect and exclude anomalous or malicious parameters.

In practice, multi-Krum first computes pairwise distances between client updates and excludes a certain fraction of updates that are farthest from the majority (likely malicious). Specifically, in the *e*-th epoch, the distances $d_{c}^{e}$ between the parameter updates from client *c* and those from all other clients is calculated as:

(7) $$d_{c}^{e}=\sum_{i=1,i\neq c}^{C}{∥\hat{\Theta_{c}^{e}}-\hat{\Theta_{i}^{e}}∥}^{2}.$$

The |bC| clients with the smallest distance sums $d_{c}^{e}$ are selected to form an initial trusted client set S for aggregation, where *b* is the Krum aggregation ratio and 0<b<1. The set of anomalous clients B is excluded. We then calculate the aggregated global model parameters as:

(8) $$\Theta_{g}^{e}=\frac{1}{\left|S\right|}\sum_{c\in S}{\hat{\Theta }}_{c}^{e}.$$

Since multi-Krumeffectively identifies and removes extreme updates that deviate significantly from the majority of updates, it ensures that the global model remains structurally and functionally robust within a certain threshold of Byzantine client proportions (typically assuming |B|<(C−2)/2C [28]).

Subsequently, on each model parameter dimension, the trimmed mean algorithm removes extreme values and averages the rest. Specifically, let the set of parameter updates for the *m*-th layer from all clients in round *e* be denoted as $U_{m}^{e}=\left\{{\hat{\Theta }}_{1,m}^{e},\ldots ,{\hat{\Theta }}_{C,m}^{e}\right\}$. After sorting $U_{m}^{e}$ by magnitude, the largest and smallest updates are removed, and the remaining parameters are averaged. This combined approach improves robustness by filtering out poisoned updates before they can corrupt the global model, thus preserving the accuracy of the output measurements.

#### 4.1.3. Sliding Average Filtering and Central Server Fine-Tuning for Backdoor Mitigation

Backdoor attacks typically implant specific trigger patterns into some clients’ training data, causing the global model to learn incorrect load mappings when those triggers appear. To combat this, we apply a sliding average filter as a denoising pre-processing step on the time-series power data at each client. Given a time-series segment, we use a sliding window of size *T* to smooth out short spikes or anomalies. If a power sequence $\tilde{p}$ contains a sudden spike (the backdoor trigger), the sliding average significantly attenuates this high-frequency signal. This prevents the model from learning the malicious mapping associated with the trigger, thereby greatly reducing the backdoor’s success ratio at the data source.

It should be noted that sliding average filtering essentially acts as a low-pass operation that is more sensitive to short-term, high-amplitude anomalies, and is therefore mainly targeted at the rectangular pulse-type backdoor triggers constructed in the simulations of this paper. In attacker-crafted stealthy trigger scenarios (e.g., triggers with low amplitude, slow variation, or those deeply embedded into normal consumption patterns), a single data-filtering step cannot guarantee complete removal of the backdoor pattern; its primary role is to attenuate the impact of typical salient triggers on the local training process.

After the model aggregation, we perform a brief fine-tuning on the global model using a small batch of clean data at the server. Typically, 1–3 epochs of fine-tuning on clean NILM samples $D_{g}$ are sufficient. This fine-tuning step effectively dilutes or removes backdoor mappings that may have been inserted into the model. Importantly, if no backdoor is present, this fine-tuning reinforces the normal load disaggregation mapping, preserving model performance. In summary, combining data filtering and server-side fine-tuning provides a robust defense against backdoor attacks without sacrificing the model’s accuracy.

#### 4.1.4. Discussion on Joint Attacks and Trade-Offs

In practice, an adversary may combine a stealthy backdoor with small-magnitude parameter perturbations to evade distance-based screening. Although we evaluate Byzantine and backdoor defenses separately for clarity, our defense pipeline is designed to be composable. Robust aggregation (multi-Krum followed by trimmed mean) operates in the update space and suppresses anomalous or poisoned uploads before they dominate the global model, whereas the client-side sliding average filtering attenuates high-frequency trigger patterns at the data source and the brief server-side fine-tuning on a small clean set further dilutes residual backdoor mappings. Under joint attacks, these components provide complementary protection across both parameter and data pathways. This also implies a robustness–performance trade-off: overly aggressive screening may remove informative but heterogeneous client updates, and stronger smoothing/fine-tuning may slightly reduce the fidelity of legitimate transients. Therefore, we keep the filtering window and fine-tuning budget small (typically 1–3 epochs) to balance robustness and disaggregation accuracy. As future work, we plan to investigate more robust joint aggregation-and-detection schemes tailored to combined attack patterns and to conduct systematic evaluations under such composite threat models.

### 4.2. GAT-Based Method for Defending Against Topology Poisoning Attacks in the DFL Mode

When operating in DFL mode, clients exchange parameters via peer-to-peer communication, exposing the system to topology poisoning attacks. Malicious clients may falsify neighbor information, spreading contaminated updates. To mitigate these threats, we adopt a dynamic topology construction approach based on GAT [38]. Formally, the communication topology is represented as an undirected graph G=(C,E), where C={1,2,…,C} is the set of clients and E⊆C×C represents the client connections. Unlike fixed or random topologies vulnerable to persistent attacks [30], our method uses GAT to dynamically select trustworthy neighbors by computing credibility weights. This creates a sparse, reliable topology, restricting malicious parameter propagation.

Specifically, each client *c* constructs a reliability feature vector $x_{c}\in R^{n}$, defined as: $x_{c}=\left[\mu_{c}^{e},\sigma_{c}^{e},{\mathrm{Abs}}_{c}^{e},{\mathrm{Max}}_{c}^{e},{\mathrm{Min}}_{c}^{e},\nu_{c}^{e}\right]$, where $\mu_{c}^{e}$ is the mean parameter change, $\sigma_{c}^{e}$ is the variance of parameter change, ${\mathrm{Abs}}_{c}^{e}$ is the mean absolute change, ${\mathrm{Max}}_{c}^{e}$ and ${\mathrm{Min}}_{c}^{e}$ denote the maximum and minimum changes, respectively, and the norm $\nu_{c}^{e}$ is calculated as: $\nu_{c}^{e}={∥\theta_{c}^{e}-\theta_{c}^{e-1}∥}_{2}$. These feature vectors from all clients are combined into a feature matrix $X={\left[x_{1},\ldots ,x_{c},\ldots ,x_{C}\right]}^{⊤}$. GAT then computes attention coefficients $\alpha_{c,u}$ for edges (c,u), quantifying client similarity and credibility. Higher coefficients indicate more reliable parameter exchanges. The overall procedure is summarized in Algorithm 1.    
**Algorithm 1:** GAT-based communication topology
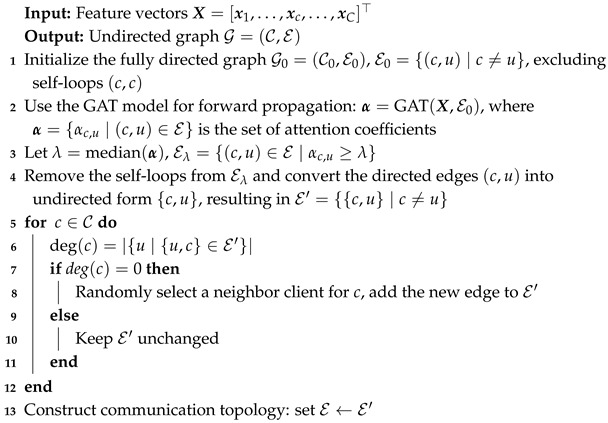


In Algorithm 1, step 2 computes the GAT attention coefficients α, reflecting client similarity. Steps 3–4 remove redundant or weak connections, retaining only edges with attention coefficients exceeding the median threshold λ, thus preserving significant connections. Steps 5–13 ensure that each client retains at least one neighbor to avoid isolated nodes. This dynamic selection method enables GAT to filter out low-trust connections and weak links based on the threshold λ, thereby restricting the propagation of malicious parameter updates. Moreover, periodic topology reconstruction (every *E* rounds) further disrupts sustained stealth attacks, increasing attacker difficulty and limiting attack duration. To avoid triggering unnecessary reconstructions due to short-term noise, the server monitors a global validation error indicator only at periodic checkpoints (every several communication rounds) and compares it with the recent stable level. A GAT-based dynamic topology reconstruction is activated only when a clear degradation of this indicator beyond a small tolerance has been observed and has persisted over multiple consecutive checkpoints, so that the communication graph is updated only in response to sustained and non-trivial performance drops rather than transient fluctuations. Through these strategies, the proposed method effectively defends against topology poisoning attacks, enhancing measurement privacy-preservation and robustness of the training process in DFL.

### 4.3. Complexity Analysis of Hybrid FL

To facilitate the complexity analysis, this paper uses the symbols listed in Table 1 to denote key variables. Subsequently, the theoretical time complexity and communication overheads for each critical module per training round, as undertaken by clients and the server, are analyzed separately. The results are summarized in Table 2, and a detailed complexity analysis is provided in Appendix A.

Additionally, the communication complexity varies depending on the federated learning mode. In CFL mode, each client transmits approximately (1−a) proportion of model parameters per round, and thus, the communication overhead scales linearly with the number of clients. In contrast, in DFL mode, each client exchanges parameters with an average of *d* neighboring clients, resulting in a communication complexity linearly related to *d* per client.

## 5. Simulation Evaluation

This section evaluates the performance of the proposed hybrid FL framework for NILM through comprehensive simulation experiments. Two widely used public NILM datasets, REFIT [39] and UK-DALE [40], are employed to verify the effectiveness of the framework. It should be noted that our previously proposed TransDisNILM model [8] is utilized as the base NILM model due to its strong representation capability in capturing temporal load features. Specifically, TransDisNILM integrates convolutional and pooling layers for preliminary feature extraction, a positional encoding mechanism, a 3-layer Transformer encoder for modeling long-range dependencies, and fully connected layers for load power disaggregation. The following subsections detail the datasets and preprocessing methods, describe the evaluation metrics, and subsequently present the experimental setup alongside the corresponding results and analyses.

### 5.1. Dataset and Preprocessing

We evaluate on two widely recognized public NILM datasets [39,40]:

(i) REFIT: Collected from 20 UK homes (2013–2015) with an 8 s sampling interval for both aggregate mains and individual load power data.

(ii) UK-DALE: Collected from 5 UK homes (2013–2015). Aggregate mains power is sampled every 1 s, and individual load every 6 s.

In our simulations, we focus on the washing machine as the target load. On the one hand, the washing machine is widely recognized in NILM research as a typical complex load [41]: its operating cycle consists of several distinct phases, and its power profile exhibits pronounced non-stationarity and frequent multi-state transitions, which makes it more difficult to disaggregate than steady-state loads such as refrigerators. On the other hand, in our previous work on transfer learning for NILM [8], we verify the effectiveness of using the washing machine as a source task and transferring to other loads, which indicates that the washing machine is highly representative of other loads in the feature space. In addition, in Appendix B we further select a kettle load, which is clearly different from the washing machine, to perform simulations and validate the effectiveness of the proposed method. Since REFIT and UK-DALE have different sampling rates, we uniformly resampled both to 0.125 Hz (one sample per 8 s). To reduce variance between households and aid model generalization, we also standardized the power values in each dataset (by subtracting the mean and dividing by the standard deviation of the aggregate power).

To simulate realistic data distributions, datasets are partitioned into 10 FL clients (see Table 3). It is important to highlight that only household 1 from UK-DALE provided sufficiently complete and continuous measurement records suitable for analysis; hence, we select only this household’s data from UK-DALE. Each client’s data is split 8:2 for training and testing, with Client 1’s test set used for final evaluation.

### 5.2. Evaluation Metrics

We adopt three metrics [42,43] to quantify performance:

(i) Mean Absolute Error (MAE): Average absolute difference between predicted ${\hat{p}}_{l}\left(t\right)$ and actual $p_{l}\left(t\right)$:

(9) $${\mathrm{MAE}}_{l}=\frac{1}{T}\sum_{t=1}^{T}\left|{\hat{p}}_{l}\left(t\right)-p_{l}\left(t\right)\right|.$$

(ii) Normalized Signal Aggregate Error (SAE): Defined as relative error of total energy consumption across the entire monitoring period:

(10) $${\mathrm{SAE}}_{l}=\left|\sum_{t=1}^{T}\left({\hat{p}}_{l}\left(t\right)-p_{l}\left(t\right)\right)\right|/\sum_{t=1}^{T}p_{l}\left(t\right).$$

(iii) Energy per Day (EpD): Daily absolute error in energy prediction:

(11) $${\mathrm{EpD}}_{l}=\frac{1}{D}\sum_{d=1}^{D}\left|\sum_{t=d\cdot \Delta T}^{(d+1)\Delta T-1}\left({\hat{p}}_{l}\left(t\right)-p_{l}\left(t\right)\right)\right|,$$

where $D=T\Delta t/\left(24\times 3600\right)$ represents the total number of days in the sample, with Δt as the sampling interval. ΔT=3600/Δt denotes the number of sampling points in a day.

### 5.3. Performance Verification

This section systematically verifies the robustness of the proposed hybrid FL framework through multiple simulations, examining its defensive performance against various attack scenarios in NILM tasks.

#### 5.3.1. Defense Against Model Inversion Attacks

(i) Analysis of Defense Methods: Model inversion attacks, such as gradient inversion [22] and GAN-based approaches [21], depend on assumptions: known complete model structures, full parameter access, and strong gradient-data correlations.

Our layer-sensitivity pruning strategy weakens these assumptions in three ways: First, dynamic pruning of sensitive parameters creates incomplete, heterogeneous uploads, disrupting attackers’ ability to anticipate parameter subsets. Prior studies [22,31,44] confirm significantly degraded reconstruction quality when pruning exceeds certain thresholds. Second, parameter incompleteness from pruning leads to irreversible information loss, creating underdetermined optimization problems and trapping attackers in local optima. Finally, pruning parameters weakens gradient-data correlations, impairing attackers’ reconstruction capabilities. Thus, our strategy effectively disrupts critical conditions necessary for successful inversion attacks.

(ii) Impact of Pruning Ratio on Model Performance: To evaluate the effectiveness and performance of our layer-sensitivity pruning strategy within the FL environment, we conducted simulations using the TransDisNILM model in a CFL setup. We investigated pruning ratios a∈{0.1,0.2,0.3}, as suggested by [45,46], assessing their impact on disaggregation performance. The results are summarized in Table 4.

From Table 4, we observe that performance degradation remains within acceptable limits (less than 10%) at pruning ratio up to 0.2, maintaining good stability and robustness. However, performance sharply declines at a pruning ratio of 0.3, highlighting significant deterioration in model accuracy. Thus, balancing model accuracy and measurement privacy-preservation, the optimal pruning ratio is approximately 0.2.

**Figure 2:**
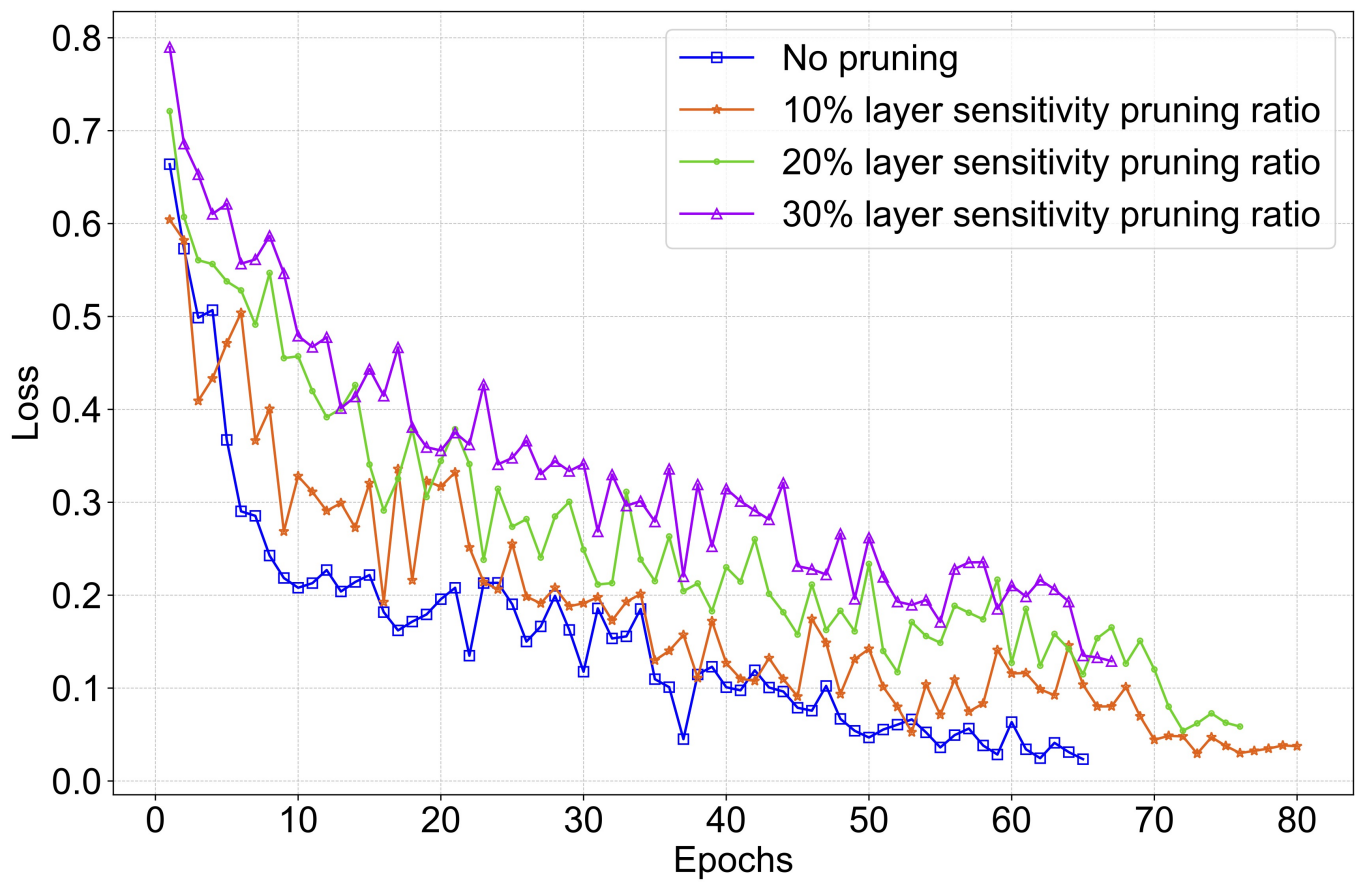
Loss curve trends for different layer-sensitivity pruning ratios in TransDisNILM.

Furthermore, Figure 2 shows convergence curves under various pruning ratios, indicating that moderate pruning (around 0.2) offers acceptable training stability and optimization robustness, whereas higher pruning ratios impair global optimization capability.

#### 5.3.2. Defense Against Parameter Poisoning Attacks

Parameter poisoning includes Byzantine and backdoor attacks. We evaluated defenses against these attacks separately.

(i) Defense Against Byzantine Attacks: To validate our robust aggregation method, simulations compared its performance against multi-Krum and trimmed mean under varying Byzantine client proportions (10%, 20%, 30%) [28,32] (see Table 5). Two attack scenarios are simulated as follows [47,48]: Gaussian noise injection attack, where malicious client updates ${\hat{\Theta }}_{c}^{e}$ are perturbed by adding element-wise i.i.d. Gaussian noise $\epsilon ∼N\left(0,\sigma^{2}\right)$ with σ=0.1 (thus Var(ϵ)=0.01), resulting in ${\tilde{\Theta }}_{c}^{e}={\hat{\Theta }}_{c}^{e}+\epsilon$; Extreme-value deviation attack, where malicious clients overwrite the uploaded updates by setting all parameter entries to a constant extreme value (1000), i.e., ${\tilde{\Theta }}_{c}^{e}=1\cdot 1000$, to induce severe deviations from normal update distributions.

From Table 5, at 10% malicious clients, all three aggregation methods demonstrate similarly strong defense capabilities. However, as the malicious client ratio increases to 20%, the trimmed mean method’s performance noticeably declines, suggesting vulnerability due to its reliance on simply excluding extreme updates. Multi-Krum provides improved robustness compared to trimmed mean but still experiences degradation. In contrast, our proposed method significantly outperforms both baselines, showing smaller increases in error metrics and better resistance to moderate attacks. At 30% malicious clients, both trimmed mean and multi-Krum suffer severe performance deterioration, with MAE and SAE increases exceeding 30%, whereas our method maintains superior robustness, limiting increases to under 15.4%.

(ii) Defense Against Backdoor Attacks: To evaluate the effectiveness of data-level filtering and server-side fine-tuning methods against backdoor attacks, we conduct targeted simulation experiments. The experiments utilize the CFL mode with the TransDisNILM model. Specifically, 30% of clients are set as malicious, randomly injecting backdoor triggers into 30% (according to reference [49,50]) of their local data segments. The injected backdoor pattern follows reference [23], characterized by a rectangular pulse with 5000 W lasting for 10 sampling points, reducing the contaminated load power abnormally to 1200 W.

**Figure 3:**
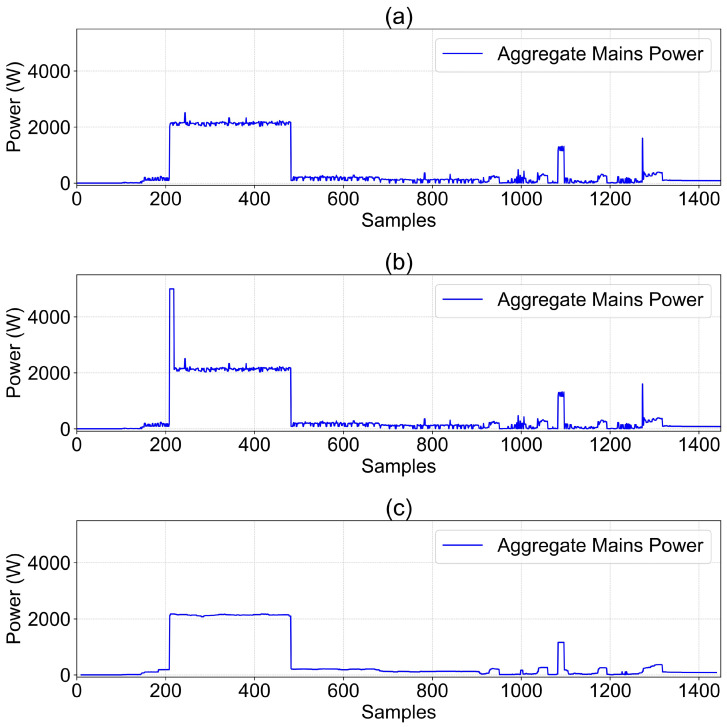
Comparison of aggregate mains power. (**a**) True value. (**b**) True value under backdoor attack. (**c**) Filtered true value under backdoor attack.

A sliding average filter is applied to the mains power, and the results are presented in Figure 3. The waveform in Figure 3a is stable without prominent spikes. In Figure 3b, a clear abnormal pulse of 5000 W appears due to the injected backdoor trigger, which is effectively smoothed and diluted by the filter in Figure 3c, reducing its magnitude by approximately 60%. The waveform after filtering closely resembles normal operational power consumption.

**Figure 4:**
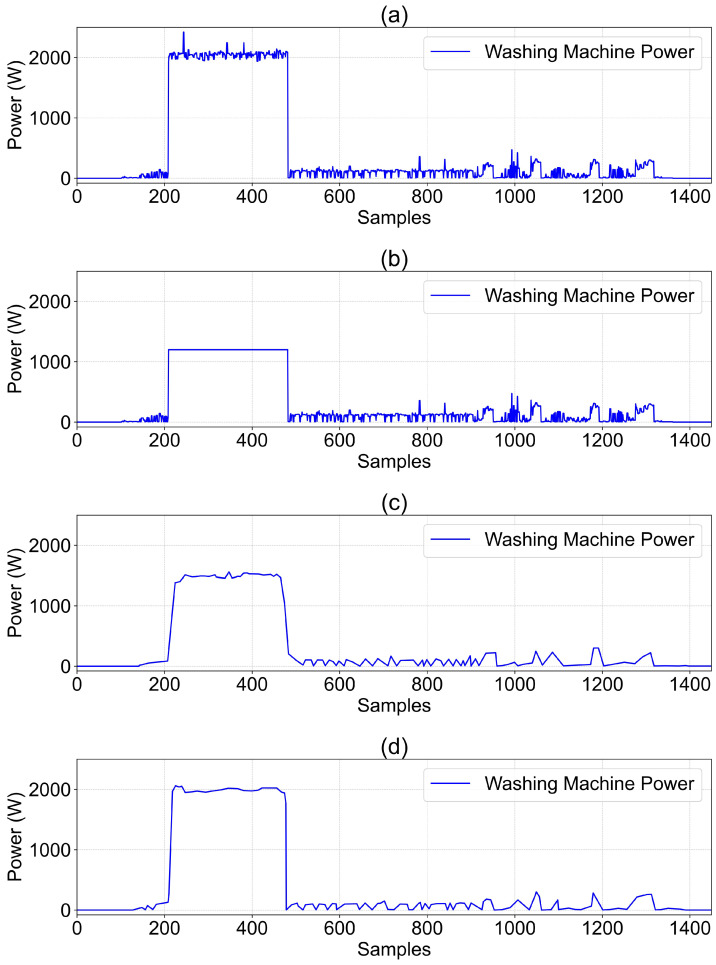
Data defense against backdoor attacks (client 1’s washing machine). (**a**) The true value. (**b**) The true value under backdoor attack. (**c**) The predicted value without fine-tuning. (**d**) The predicted value after fine-tuning.

Further, the aggregated model undergoes fine-tuning using a small batch of clean data (1000 sample points), with a fine-tuning duration of 3 epochs and a learning rate of 0.0001. Performance before and after fine-tuning is compared in Table 6. The server-side fine-tuning significantly improves all three performance metrics, enhancing each by over 30%. This demonstrates the efficacy of mini-batch fine-tuning in effectively removing backdoor mappings from the aggregated model. Figure 4 further visually illustrates prediction waveforms before and after fine-tuning. As shown, the non-tuned model exhibits pronounced prediction anomalies, while predictions from the fine-tuned model appear more stable, confirming the effectiveness of the proposed defense method.

#### 5.3.3. Defense Against Topology Poisoning

**Figure 5:**
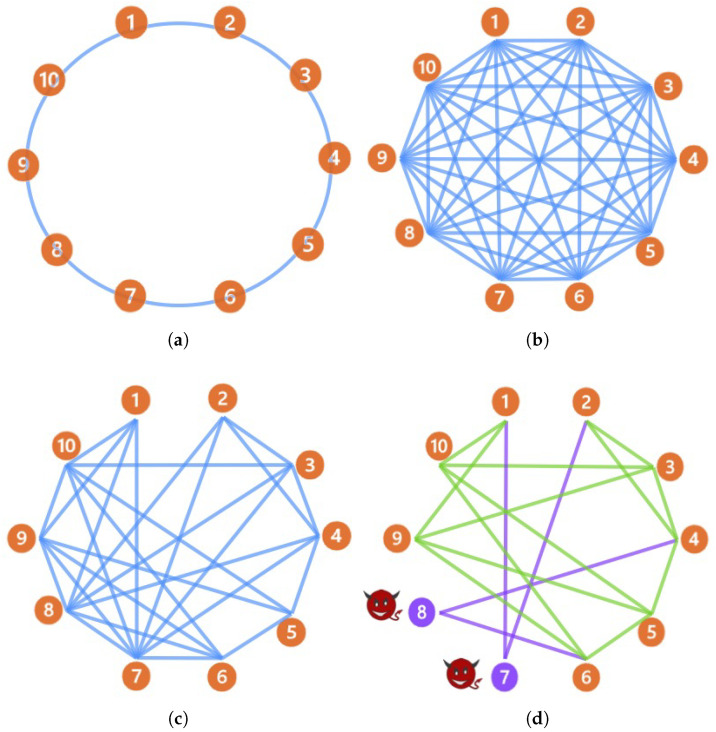
Schematic diagram of three communication topologies in DFL mode. (**a**) Ring-connected topology. (**b**) Fully connected topology. (**c**) GAT-connected topology. (**d**) The reconstructed topology of GAT after topological poisoning. The numbers represent different clients, and the purple color indicates that the client is under attack.

(i) Performance Comparison of Different Communication Topologies: When the central server fails, this study investigates the impact of different communication topologies on model performance and convergence speed under DFL mode. As illustrated in Figure 5a–c, three representative topologies are selected for comparison. The first is the fully connected topology, where all clients directly communicate with each other, as shown in Figure 5a. The second is the ring-connected topology [30], in which clients form a ring structure and communicate only with their immediate neighbors, as depicted in Figure 5b. The third is the GAT-connected topology, illustrated in Figure 5c, where connections are dynamically established based on client feature similarity using GAT.

**Figure 6:**
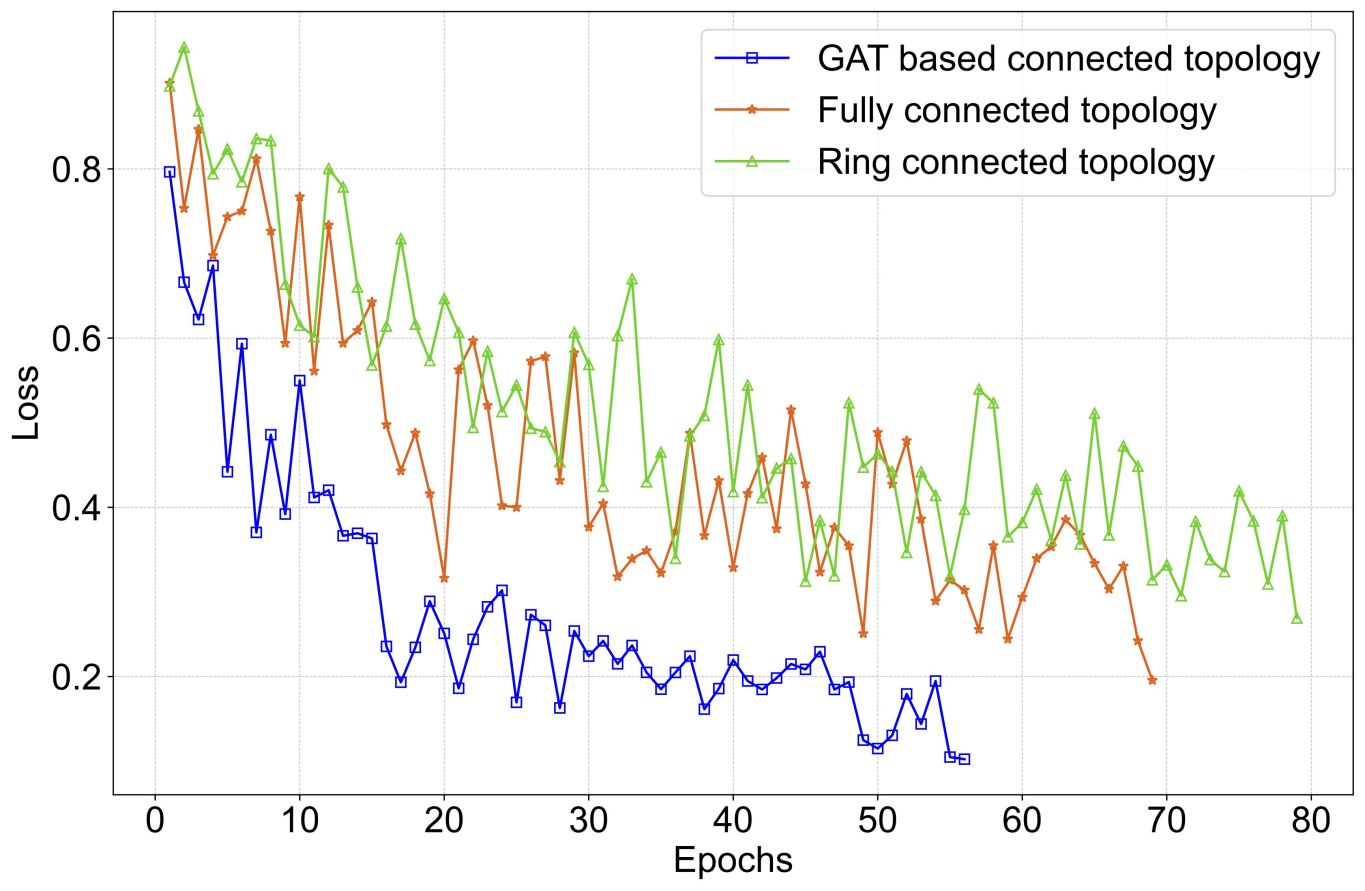
Loss curves for TransDisNILM during DFL with different topologies.

Simulation experiments using the TransDisNILM model are conducted under these topologies in DFL mode. Results are presented in Table 7, with corresponding training loss curves shown in Figure 6. From Table 7, it is evident that the GAT-connected topology achieves better performance in all evaluation metrics. Figure 6 further confirms this advantage, showing faster convergence rates and lower loss values compared to the other two topologies. These results validate the efficacy of the GAT-connected topology in enhancing collaboration efficiency within DFL mode.

The observed results can be explained as follows: although the fully connected topology theoretically offers comprehensive information exchange, excessive redundant transmission may slow model convergence. The ring-connected topology incurs lower communication overhead; however, the extended communication chain restricts convergence speed and limits model performance. Conversely, the GAT-connected topology adjusts connections based on client similarities and data distributions, effectively reducing unnecessary parameter transmissions and enhancing meaningful information exchanges. As a result, this approach improves both learning efficiency and overall model performance.

(ii) Topology Poisoning Attack and GAT Defense Verification: To further validate the effectiveness of the proposed GAT-connected topology reconstruction method against topology poisoning attacks in DFL mode, we design detailed attack scenarios and simulations. Initially, GAT constructs the communication topology among ten clients (Figure 5c). Analysis of degree distributions identifies clients 7 and 8 as critical nodes, having the highest degrees (8 and 7, respectively).

These two clients are set as malicious attackers conducting topology poisoning attacks by broadcasting extremely deviating parameters to maximize contamination spread. Following the attack, model performance significantly deteriorates, as seen in the second-to-last row of Table 7, with SAE deteriorating over 41%. When performance degradation is detected, we activate the GAT dynamic topology reconstruction method (Algorithm 1). It effectively isolates malicious clients 7 and 8 (Figure 5d). Post-defense performance rapidly recovers, with MAE decreasing from 35.203 W to 29.156 W, effectively mitigating the attack’s impact on the measurement accuracy of the model. Overall, the simulations confirm the robustness of GAT-connected dynamic topology reconstruction, highlighting its ability to intelligently detect and isolate malicious clients, significantly improving security and robustness in DFL.

## 6. Conclusions

This paper has proposed a hybrid FL framework to address the significant privacy leakage risks associated with traditional NILM methods in centralized training modes. The framework has the capability to switch adaptively between CFL and DFL modes depending on the availability of the central server. Additionally, robust defense methods have been designed to counter model inversion attacks, parameter poisoning attacks (including Byzantine and backdoor attacks), and topology poisoning attacks.

In CFL mode, our layer-sensitivity pruning method effectively reduces the invertibility of model parameters and mitigates model inversion attacks. In our tests, a 20% pruning ratio kept the load disaggregation measurement accuracy loss under 10%. Furthermore, the combination of multi-Krum and trimmed mean robust aggregation with sliding average filtering and server-side fine-tuning effectively defended against parameter poisoning. Even with 30% malicious clients, the framework kept performance degradation below 15.4%. It should be noted that for more stealthy low-frequency or pattern-similar triggers, this work mainly focuses on empirically enhancing robustness through a multi-layer defense chain (data filtering, robust aggregation, and server-side fine-tuning), while more systematic detection methods and theoretical analysis are left as important directions for future work. In DFL mode, the proposed GAT-based dynamic topology construction method has proactively isolated highly connected malicious clients, rapidly restoring model performance to normal levels after an attack (approximately 17.2% reduction in MAE), thus effectively preventing continuous topology poisoning attacks.

Overall, our hybrid FL framework significantly improves smart grid NILM security and privacy. It enhances the robustness and reliability of power system measurements against privacy threats and various typical attacks. Consequently, it provides a theoretical foundation for smart grid measurement research. Future work will further conduct more extensive validation on a wider range of load types and a larger user population. In addition, we plan to develop joint aggregation-and-detection schemes tailored to combined attack patterns and to evaluate them systematically under composite threat models.

## Figures and Tables

**Table 1:** Notation of key variables.

No.	Notation	Description
1	*C*	Number of clients participating in FL
2	*P*	Total number of model parameters
3	*M*	Number of layers in the model
4	*a*	Pruning ratio for layer-sensitive pruning (0<a<1)
5	*N*	Number of local training samples per client
6	*G*	Size of the clean dataset for fine-tuning (G≪N)
7	*e*	Number of fine-tuning epochs
8	*n*	Dimension of the feature vector in the GAT method
9	*d*	Average number of neighbors connected per client in DFL

**Table 2:** Complexity analysis of each module.

No.	Module	Execution	Time Complexity	Communication Overhead (per Round)
1	Layer-sensitive pruning	client	O(P+MlogM)	Upload: (1−a)P; Download: *P*
2	Sliding average filtering	client	O(N)	None
3	Multi-Krum aggregation	server	$O(C^{2}\left(1-a\right)P)$	None
4	Trimmed Mean aggregation	server	O(PClogC)	None
5	Server-side fine-tuning	server	O(eGP)	None
6	GAT-based topology	client/server	$O(C^{2}n+C^{2}\log C)$	When topology update. Upload: O(n); Download: topology table per client: O(d)

**Table 3:** Data partitioning for federated learning clients.

Client ID	Dataset	Household ID	Training Set (MB)	Test Set (MB)
1	REFIT	2	26.05	6.51
2	REFIT	5	35.70	8.93
3	REFIT	7	32.17	8.04
4	REFIT	8	28.97	7.24
5	REFIT	9	28.97	7.24
6	REFIT	15	28.82	7.21
7	REFIT	16	26.83	6.71
8	REFIT	17	25.05	6.26
9	REFIT	18	23.01	5.75
10	UK-DALE	1	63.03	15.76

**Table 4:** Comparison of TransDisNILM model performance under different pruning ratios.

Pruning Ratio *a*	MAE (W)	SAE	EpD (Wh)
0 (No Pruning)	24.863	0.164	168.540
0.1	25.446 (+2.3%)	0.169 (+3.0%)	182.134 (+8.1%)
0.2	27.265 (+9.7%)	0.171 (+4.3%)	185.900 (+10.3%)
0.3	33.957 (+36.6%)	0.218 (+32.9%)	201.748 (+19.7%)

Note: Percentages represent degradation relative to the unpruned model.

**Table 5:** Robust aggregation performance under varying Byzantine client ratios.

Malicious Ratio	Aggregation Method	MAE (W)	SAE	EpD (Wh)
10%	Multi-Krum	28.286	0.188	191.495
	Trimmed Mean	28.074	0.184	189.481
	This paper	27.439	0.172	183.120
20%	Multi-Krum	31.856	0.209	194.399
	Trimmed Mean	34.604	0.233	213.239
	This paper	27.866	0.179	189.275
30%	Multi-Krum	38.258	0.247	227.656
	Trimmed Mean	42.115	0.316	273.345
	This paper	31.447	0.198	190.258

**Table 6:** Model performance comparison before and after fine-tuning.

Fine-Tuning	MAE (W)	SAE	EpD (Wh)
No	39.174	0.309	232.429
Yes	22.667 (+42.1%)	0.160 (+48.2%)	158.908 (+31.6%)

Note: Percentages in parentheses indicate performance improvements relative to the baseline (no fine-tuning).

**Table 7:** Model performance under different communication topologies in DFL mode.

Topology	MAE (W)	SAE	EpD (Wh)
Fully Connected	29.401	0.198	201.621
Ring-Connected	37.385	0.223	246.702
GAT-Connected	27.367	0.178	193.029
Poisoned clients 7 and 8 (no defense of GAT-connected)	35.203	0.251	228.317
Poisoned clients 7 and 8 (after defense of GAT-connected)	29.156	0.195	196.434

## Data Availability

To access the public dataset used in this paper, please follow the links provided: https://pureportal.strath.ac.uk/en/datasets/refit-electrical-load-measurements-cleaned/ (accessed on 8 January 2026), for REFIT, and https://data.ceda.ac.uk/edc/efficiency/residential/EnergyConsumption/Domestic/UK-DALE-2015 (accessed on 8 January 2026), for UK-DALE.

## Appendix A

The following provides a detailed analysis and explanation of the complexity summarized in Table 2.

(i) **Layer-sensitive pruning:** From the perspective of time complexity, each client computes the mean value layer-by-layer for all *P* parameters per round and sorts *M* sensitivity layers to select the top (1−a)M highly sensitive layers. The overall time complexity is approximately O(P+MlogM), which can be simplified to O(P) since M≪P. As for communication and computational overhead, With pruning, each client uploads only (1−a)P important parameters per round, reducing the communication volume to (1−a) of the original. The additional computations (mean calculation and sorting) are negligible compared to local training iterations.

(ii) **Sliding average filtering:** From the perspective of time complexity, each client performs a convolution operation of length *T* on its *N* local data points, yielding an overall complexity of O(N). Filtering occurs only once during data loading and is not repeated in subsequent training rounds. As for communication and computational overhead, no extra communication overhead is generated, and the computational burden on clients is minimal.

(iii) **Multi-Krum aggregation:** From the perspective of time complexity, the server operates on the pruned update vectors of dimension (1−a)P uploaded by *C* clients. First, it calculates the pairwise distances between the update vectors of the *C* clients, with a total of C(1−C)/2 pairs, and the complexity of each pair is O((1−a)P). Therefore, the overall time complexity is $O(C^{2}\left(1-a\right)P)$. As for communication and computational overhead, multi-Krum does not introduce any extra uplink communication beyond transmitting the pruned model updates themselves; the entire robust aggregation is performed on the server side. In our community-level AMI setting, each CFL instance corresponds to a single community where all smart-meter clients participate in aggregation and the number of clients per round is typically on the order of tens to about one hundred, while the effective parameter dimension has been reduced from *P* to (1−a)P by layer-sensitive pruning. Under this regime, the server-side computational cost of multi-Krum remains acceptable for modern hardware and does not become the bottleneck of the overall training process.

(iv) **Trimmed mean aggregation:** From the perspective of time complexity, sorting for each model parameter is required, with complexity O(PClogC). As for communication and computational overhead, trimmed mean introduces no additional communication overhead. When combined with multi-Krum, pre-selection can reduce the number of participating clients, significantly lowering computational overhead.

(v) **Server-side fine-tuning:** From the perspective of time complexity, server-side fine-tuning for *e* epochs has complexity O(eGP). In practice, *e* and *G* are typically small, making this step negligible compared to client training. As for communication and computational overhead, fine-tuning is entirely executed server-side, introduces no additional communication overhead, and minimally affects the overall duration per round, thus considered negligible.

(vi) **GAT-based topology:** From the perspective of time complexity, GAT computes attention coefficients for all pairs of clients in a fully connected graph, with complexity $O(nC^{2})$, followed by median selection from C(C−1) attention coefficients requiring sorting complexity $O(C^{2}\log C)$. The total complexity is $O(nC^{2}+C^{2}\log C)$. For each client, communication complexity per round is O(dP), with d≪C. As for communication and computational overhead, during topology construction, each client uploads only a small amount of feature vector with dimension *n*. Topology updates occur every *E* rounds, occupying only a single FL round, significantly reducing communication overhead compared to a fully connected topology.

## Appendix B

To verify the applicability of the proposed defense framework to other loads, we further conduct comparative simulations on a kettle. Table A1 reports the data partitioning of the kettle among FL clients, and Table A2 records the model performance under three configurations: no attack, pruning with a=0.2, and 30% malicious ratio with the defense enabled.

**Table A1:** Data partitioning of the kettle load among FL clients.

Client ID	Dataset	Household ID	Training Set (MB)	Test Set (MB)
1	REFIT	2	27.12	6.78
2	REFIT	5	36.80	9.20
3	REFIT	7	33.04	8.26
4	REFIT	9	30.32	7.58
5	REFIT	12	28.88	7.22
6	REFIT	3	34.64	8.66
7	REFIT	4	32.24	8.06
8	REFIT	6	30.32	7.58
9	REFIT	8	30.16	7.54
10	UK-DALE	1	63.03	15.76

**Table A2:** Comparison of model performance on the kettle under different simulation settings.

Simulation Setting	MAE (W)	SAE	EpD (Wh)
No Pruning	29.571	0.452	328.754
a=0.2 pruning	33.060 (+11.8%)	0.501 (+10.9%)	359.657 (+9.4%)
30% malicious ratio	33.622 (+13.7%)	0.504 (+11.4%)	364.259 (+10.8%)

Note: Percentages represent degradation relative to the unpruned model.

We design the simulations on the kettle in Table A2 according to the configuration that performs best on the washing machine model. As shown in Table 2, similar to the results on the washing machine load, attacks also cause performance degradation for all methods on the kettle. Specifically, under the proposed pruning setting a=0.2, the degradation of MAE, SAE, and EpD is effectively controlled within 12%. In addition, under the 30% malicious ratio setting, the performance drops of these metrics are also kept within 13.7%. These results indicate that the proposed defense framework provides consistently robust gains across different types of loads.
